# Recruitment to multicentre trials—lessons from UKCTOCS: descriptive study

**DOI:** 10.1136/bmj.a2079

**Published:** 2008-11-13

**Authors:** Usha Menon, Aleksandra Gentry-Maharaj, Andy Ryan, Aarti Sharma, Matthew Burnell, Rachel Hallett, Sara Lewis, Alberto Lopez, Keith Godfrey, David Oram, Jonathan Herod, Karin Williamson, Mourad Seif, Ian Scott, Tim Mould, Robert Woolas, John Murdoch, Stephen Dobbs, Nazar Amso, Simon Leeson, Derek Cruickshank, Ali McGuire, Stuart Campbell, Lesley Fallowfield, Steve Skates, Mahesh Parmar, Ian Jacobs

**Affiliations:** 1 Gynaecological Oncology, UCL EGA Institute for Women’s Health, London W1T 7DN; 2 Queen Elizabeth Hospital, Gateshead NE9 6SX; 3 St Bartholomew’s Hospital, London EC1A 7BE; 4 Liverpool Women’s Hospital, Liverpool L8 7SS; 5 Nottingham City Hospital, Nottingham NG5 1PB; 6 St Mary’s Hospital, Manchester M13 9WL; 7 Derby City Hospital, Derby DE22 3NE; 8 Royal Free Hospital, London NW3 2QG; 9 St Mary’s Hospital, Portsmouth PO3 6AD; 10 St Michael’s Hospital, Bristol BS2 8EG; 11 Belfast City Hospital, Belfast BT9 7AB; 12 University of Wales College of Medicine, Cardiff CF14 4XN; 13 Llandudno Hospital, Llandudno LL30 1LB; 14 James Cook University Hospital, Middlesbrough TS4 3BW; 15 London School of Economics, London WC2A 2AE; 16 Create Health Clinic, London W1G 6AJ; 17 Brighton and Sussex Medical School, University of Sussex, Brighton BN1 9PX; 18 Harvard Medical School, Boston, MA 02115, USA; 19 Medical Research Council Clinical Trials Unit, London NW1 2DA

## Abstract

**Objective** To describe the factors that contributed to successful recruitment of more than 200 000 women to the UK Collaborative Trial of Ovarian Cancer Screening, one of the largest ever randomised controlled trials.

**Design** Descriptive study.

**Setting** 13 NHS trusts in England, Wales, and Northern Ireland.

**Participants** Postmenopausal women aged 50-74; exclusion criteria included ovarian malignancy, bilateral oophorectomy, increased risk of familial ovarian cancer, active non-ovarian malignancy, and participation in other ovarian cancer screening trials.

**Main outcome measures** Achievement of target recruitment, acceptance rates of invitation, and recruitment rates.

**Results** The trial was set up in 13 centres with 27 adjoining local health authorities. The coordinating centre team was led by one of the senior investigators, who was closely involved in planning and day to day trial management. Of 1 243 282 women invited, 23.2% (288 955) replied that they were eligible and would like to participate. Of those sent appointments, 73.6% (205 090) attended for recruitment. The acceptance rate varied from 19% to 33% between trial centres. Measures to ensure target recruitment included named coordinating centre staff supporting and monitoring each centre, prompt identification and resolution of logistic problems, varying the volume of invitations by centre, using local non-attendance rates to determine the size of recruitment clinics, and organising large ad hoc clinics supported by coordinating centre staff. The trial randomised 202 638 women in 4.3 years.

**Conclusions** Planning and trial management are as important as trial design and require equal attention from senior investigators. Successful recruitment needs constant monitoring by a committed proactive management team that is willing to explore individual solutions for different centres and use central resources to improve local recruitment. Automation of trial processes with web based trial management systems is crucial in large multicentre randomised controlled trials. Recruitment can be further enhanced by using information videos and group discussions.

**Trial registration** Current Controlled Trials ISRCTN22488978.

## Introduction

Randomised controlled trials are widely accepted as the standard for evaluating healthcare interventions. However, only 31% of multicentre randomised controlled trials funded by the UK Medical Research Council and the NHS Health Technology Assessment Programme that were recruiting between 1994 and 2002 achieved their original recruitment target.^1^ This has important implications for allocation of resources, lost opportunity for trials that fail to complete, and loss of statistical power for completed trials.^2^

Although the design and scientific validity of randomised controlled trials have been subject to intense scrutiny, the management and conduct of these trials receive limited attention. Large multicentre randomised controlled trials, like other complex organisational ventures, need meticulous management if they are to be successful. Many fail to deliver because of the lack of a practical professional approach to getting the job done.^3^ Furthermore, in randomised controlled trials involving screening and prevention, having to invite large numbers of potential participants is an additional challenge.^4^

In 2000 expertise accumulated over a decade of planning and running ovarian cancer screening trials was used to design and set up the UK Collaborative Trial of Ovarian Cancer Screening (UKCTOCS; www.ukctocs.org.uk).^5^ ^6^ ^7^ ^8^ ^9^ Recruitment of the required target of 202 638 women was completed in 2005, making it one of the largest ever randomised controlled trials. This report describes the approach to planning and management that contributed to successful recruitment.

## Methods

The UKCTOCS is a randomised controlled trial aiming to assess the impact of screening on mortality from ovarian cancer while comprehensively evaluating performance characteristics of the screening strategies, physical and psychological morbidity, compliance, and cost. The design involves 200 000 women randomised to annual screening with serum CA 125 or transvaginal ultrasound or no intervention (fig 1). The inclusion criteria were women aged 50-74 and with postmenopausal status; the exclusion criteria were bilateral oophorectomy, previous ovarian malignancy, increased risk of familial ovarian cancer, active non-ovarian malignancy, and participation in other ovarian cancer screening trials.

**Figure fig1:**
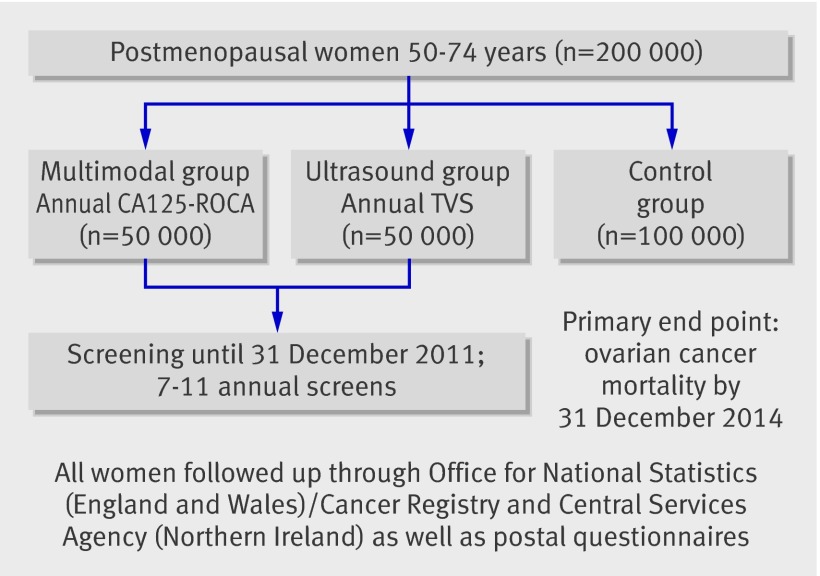
**Fig 1** Trial design of UK Collaborative Trial of Ovarian Cancer Screening. ROCA=risk of ovarian cancer algorithm; TVS=transvaginal scan

A senior investigator leads trial management based at the coordinating centre. We use a custom built, web based trial management system to centralise and automate trial processes. We identified NHS trusts (trial centres) wishing to participate and set them up in a staggered fashion over the course of two years. Recruitment started at a trial centre when at least 1500 local women had accepted the invitation. The launch of the trial was accompanied by national media coverage. This was followed by local media coverage in the form of radio interviews and newspaper articles as each centre started recruitment. We briefed the staff manning the telephone lines of the patient support charities OVACOME and Cancer BACUP and provided them with answers to frequently asked questions before any publicity.

### Invitation

We sent information about the trial to all general practitioners working in participating primary care trusts, after electronic upload of their details into the trial management system. We requested electronic files containing details of 2000 to 10 000 women on a regular (usually three monthly) basis from each of the participating primary care trusts for upload. We then sent women personal invitations and logged replies on the trial management system (fig 2). The patient support groups OVACOME and Cancer BACUP vetted the information for patients, and invitation letters contained their contact details. In the course of the trial, we revised and simplified the invitation.

**Figure fig2:**
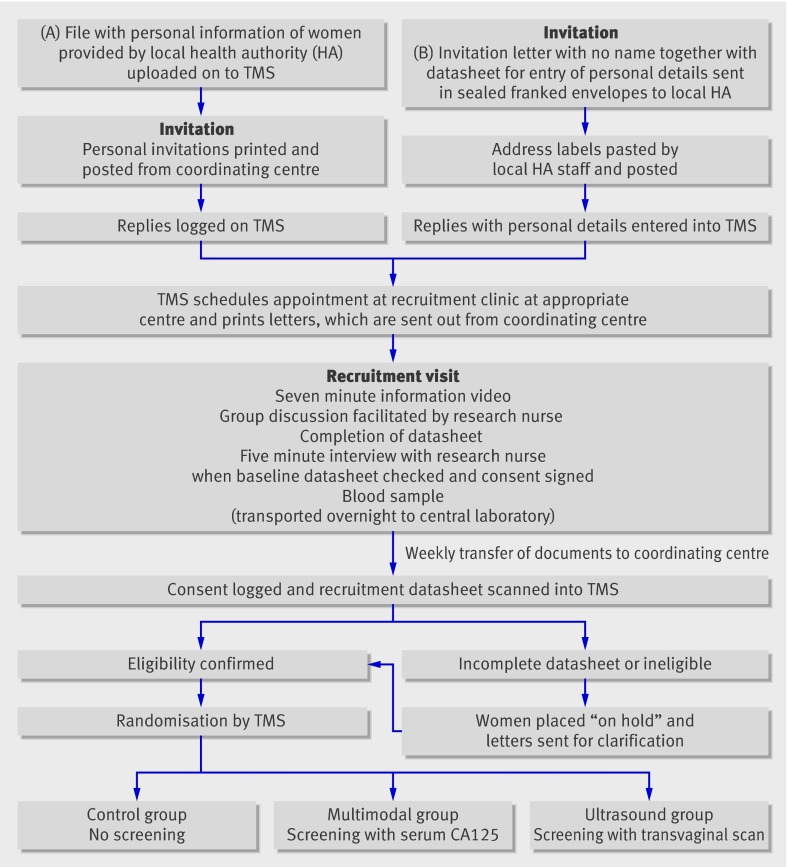
**Fig 2** Invitation, recruitment, and randomisation. (A)=recruitment in which primary care trusts (PCTs) were allowed access to contact details of women; (B)=recruitment in which PCTs did not allow access to contact details of women; TMS=trial management system

### Recruitment and randomisation

We set the weekly recruitment target at 100 women per trial centre. We set up individual profiles comprising five recruitment clinics a week on the trial management system for each trial centre. These consisted of 45 minute appointments involving groups of four women. Once acceptance was logged, women were automatically scheduled into the appointment slots at the centre and sent letters. The web browser enabled immediate logging of clinic attendance as well as rescheduling of appointments by both local and coordinating centre staff. At recruitment, women viewed an information video and participated in a group discussion. This was immediately followed by a one to one discussion in a separate room with the research nurse, when women were given the opportunity to discuss any private concerns before signing consent. The nurse also checked their completed datasheet. These documents were sent weekly to the coordinating centre, where the trial management system automatically confirmed eligibility, did randomisation, scheduled appropriate appointments to women allocated to screening, and printed letters to the patient and her general practitioner (fig 2). We sent requests for more information to women with incomplete data and placed “on hold” those whose last menstrual period was less than 12 months from date of recruitment. The trial management system also classifies screening results (ultrasound findings and CA 125 concentrations) entered over the web browser, schedules appropriate follow-up appointments, and prints all letters to individual women. It allows electronic exchange of information with the CA 125 analyser in the laboratory and with the Office for National Statistics.

We logged and monitored all complaints centrally. A designated person at the coordinating centre wrote to each woman directly after investigating the problems raised. We explored all suggestions by the women and amended trial logistics when appropriate and possible.

## Results

Between April 2001 and February 2003 we set up the trial in 13 NHS trusts (trial centres) in England, Wales, and Northern Ireland (table[Table tbl1]). A variety of logistical reasons (lack of space, retirement of potential research leads, unwillingness of trusts’ management to commit to a 10 year trial, involvement in other ovarian cancer screening trials) prevented pre-identified Scottish centres from participating. Overall, 27 primary care trusts (including local health boards in Wales) with 3266 general practices were involved. Of these, 22 (81.5%) primary care trusts provided contact details of the women, and we sent invitations as outlined above. Five (18.5%) primary care trusts (those adjoining the centres at Liverpool, Belfast, and initially Gateshead) refused access to the contact details. For these trusts, we negotiated an alternative method using “unlinked” invitations. This involved sending the standard invitation letter without a recipient’s name to the primary care trust in sealed franked envelopes. Trust staff then attached address labels and forwarded the letters. Women who wished to participate wrote to the coordinating centre with their contact details (fig 2). We modified trial processes and the trial management system to allow manual entry of these data.

**Table tbl1:** Acceptance and recruitment rates at each regional centre

Regional centre	Invited	Accepted	Reported they were ineligible*	Acceptance rate† (%)	No randomised	No of active years	No randomised per week
Manchester	133 584	26 027	8 939	20.9	16 504	3.3	114
Derby	65 391	19 688	5 093	32.7	14 920	3.0	113
Cardiff	96 633	23 890	8 754	27.2	16 756	3.4	112
Portsmouth	95 004	27 462	8 743	31.8	19 182	4.0	109
East London (Bart’s)	167 419	30 047	10 273	19.1	19 944	4.2	108
North Wales	73 956	21 299	5 144	31.0	14 326	3.3	99
North London (Royal Free)	133 572	24 391	6 416	19.2	16 737	3.9	98
Bristol	75 016	22 863	5 706	33.0	16 550	3.9	96
Nottingham	77 052	21 906	7 001	31.3	16 779	4.2	91
Gateshead	109 026	24 635	8 882	22.9	17 323	4.4	89
Gateshead (PCT invited)‡	13 000	1 323					
Middlesbrough	52 832	13 569	3 274	27.4	9 926	2.6	87
Belfast (PCT invited)‡	86 810	16 962	NA	19.5	13 579	3.6	86
Liverpool (PCT invited)‡	63 987	14 893	NA	23.3	10 112	4.0	57
Overall	1 243 282	288 955	NA	24.8	202 638	47.8	96
Overall excluding Gateshead, Belfast and Liverpool PCT invited	1 079 485	255 777	78 225	25.5	NA	NA	NA

NA=not applicable; PCT=primary care trust.

*Data incomplete as unknown proportion of ineligible women did not reply.

†Ineligible women not included in numerator or denominator.

‡Only eligible women replied owing to manner in which invitations were sent,.

### Invitation

We closely monitored acceptance rates from the start and therefore noted within the first six months that recruitment was below target. We rapidly introduced measures (listed below) to improve rates of acceptance of the invitation. Overall, invitations were sent to 1 243 282 women, 1 079 485 directly by the coordinating centre and 163 797 by the primary care trusts adjoining the centres at Liverpool, Belfast, and initially Gateshead; 288 955 (23.2%) women replied that they would like to participate in the trial and were eligible. The overall acceptance rate varied between centres from 19% in East London to 33% in Bristol (table[Table tbl1]).

### Recruitment and randomisation

Between April 2001 and September 2005 205 090 women (73.6% of those who were sent appointments) attended for recruitment, and we finally randomised 202 638 women (fig 3). The number of women randomised each month ranged from 117 to 5773 (median 3955)^10^; the median time from recruitment to randomisation was 12.3 days (25th to 75th centile 8.5 to 15.5 days).

**Figure fig3:**
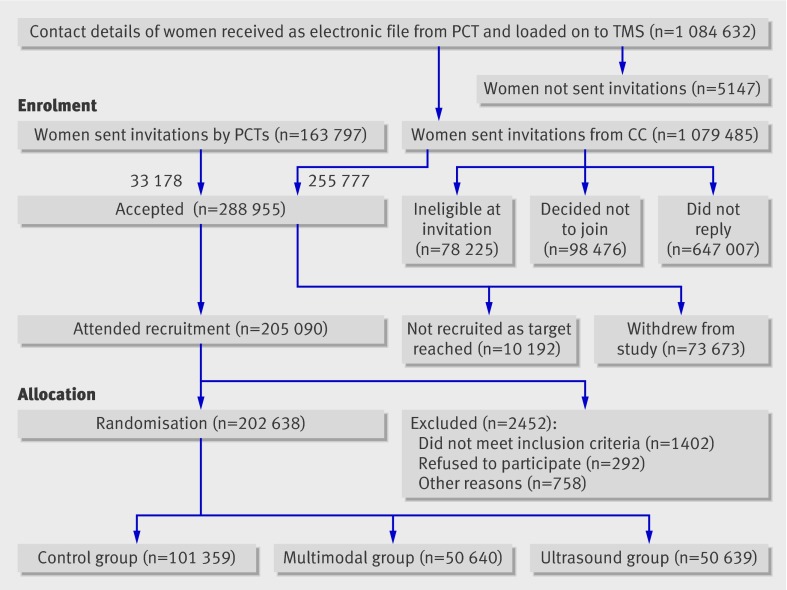
**Fig 3** Flow diagram of progress through recruitment phase of UK Collaborative Trial of Ovarian Cancer Screening. CC=coordinating centre; PCT=primary care trust; TMS=trial management system

A further 10 192 women who accepted the invitation were not sent appointments as the recruitment target was achieved. With ethics committee approval, we sent a letter to thank each woman for her interest in the trial and apologise for our inability to offer her a place as the target recruitment had been reached.

Overall recruitment was 96% (range 87-114%) of the weekly target of 100 women. Five centres exceeded the weekly target (table[Table tbl1]). The centres using “unlinked invitations” had some of the lowest recruitment rates. The box details the measures we adopted to ensure target recruitment. Ninety eight (0.008%) invited women complained about recruitment related problems: invitation to trial (32), trial information (28), recruitment appointment (17), and randomisation to control group (21).

**Strategies adopted to facilitate recruitment**

**Set-up**

- Custom built trial management system to centralise and automate all trial processes such as invitation, logging of replies, scheduling of appointments, confirmation of eligibility, randomisation, and printing of letters
- Web browser and high security encryption enabling staff at all locations to enter and view data and reschedule appointments
- Specialised software commissioned from the NHS to flag women on primary care trusts’ registers and allow electronic transfer of their personal and general practice details to the coordinating centre in lots of 5000 to 10 000 every quarter
- Trial website (www.ukctocs.org) for use by lay people and health professionals

**Invitation**

- Support of relevant patients’ groups/charities
- National and local media coverage
- Mass mailing of invitations from coordinating centre
- Personal invitation with tear-off reply slip and prepaid return envelopes
- Accompanying brochure describing the goals and requirements of the trial in simple and concise terms
- Regular monitoring of acceptance rates to establish frequency and volume of mailing needed

**Recruitment**

- Ensuring adequate numbers of women have accepted the invitation before starting recruitment at a trial centre
- Increasing appointments in individual recruitment clinics to accommodate low attendance
- Additional large ad hoc clinics staffed by both local and coordinating centre teams
- Information video at recruitment appointment
- Interactive group discussions

**Management by coordinating centre**

- Senior investigator involved in day to day running of trial
- Proactive management team
- Named coordinating centre member interacting with each trial centre on a daily basis
- Prompt identification and resolution of logistical problems (staffing, delivery of consumables, information technology networking problems, postal strikes)
- Fortnightly monitoring of targets

## Discussion

Our experience highlights the importance of meticulous planning and management of trial processes. These are as crucial to success as trial design and require equal attention from senior investigators. We quickly learnt that the key to successful recruitment was constant monitoring by a dedicated management team, capable of delivering flexible and rapid solutions as problems arose. Centralisation and automation of trial processes with web based trial management systems were crucial. Information videos and group discussions facilitated recruitment and helped to maintain quality.

The revision of the UKCTOCS invitation procedure provides an example of the flexibility needed in running trials. Although most (81.5%) local health authority data controllers allowed access to women’s contact details under the research exemption clause of the Data Protection Act 1998 (www.informationcommissioner.gov.uk), 18.5% (n=5) refused permission. We found a compromise to accommodate their concerns and revised the entire invitation process, including functionality of the trial management system and the job description of administrative staff at the coordinating centre. Excluding these health authorities would have required suspension of the trial in the adjoining centres, with a major impact on recruitment rates, time, and resources.

### Strengths and weaknesses

We invited women from participating local health authorities’ registers. This is an approach often used in the United Kingdom.^11^ ^12^ Other databases in use include electoral rolls, driving licence records, and commercially available directories.^13^ The alternative strategy is to advertise the trial extensively and let participants self refer.^14^ Recently, this has involved Google adverts and links to studies on a variety of websites.^15^ Women who volunteer to participate in research are often more educated and informed than the general population.^16^ ^17^ ^18^ Hence, invitation using health authority lists or electoral rolls is thought to result in participants who are more representative of the general population than those who self refer through adverts. Use of health authority lists provides additional logistical advantages. Recruitment rates can be controlled by varying the volume and frequency of mailing invitations, and electronic data transfer from health authority files leads to decreased data entry and improved accuracy. This is particularly notable with regard to NHS numbers, which are essential for cost effective follow-up of women through the UK Office for National Statistics. In a previous trial, in which women self referred, most women did not provide an NHS number.^19^ Finally, mass mailing allows acceptance rates and their variation between centres to be determined.

Recruitment rates vary with time and between centres. Maintaining this at target levels requires constant monitoring and individual solutions, as problems differ between centres. Support from the relevant patients’ support groups is crucial. For trials with lower acceptance rates, one solution is to increase the volume and frequency of mailing of invitations and local media coverage. Mass broadcast (television and radio) and print media are “low effort, high yield” recruitment strategies.^20^ ^21^ In the UKCTOCS, as participation was limited to invited women, we were constantly trying to find the balance between too much and too little publicity. To this end, we avoided posters and flyers in general practitioners’ surgeries, well women clinics, and so on. Such publicity may have persuaded more women to take up their invitation.

In FLEXISIG, the sigmoidoscopy screening trial, the involved general practices checked the local authority lists and excluded 2% of 375 744 men and women as they were deemed ineligible owing to colorectal cancer, terminal disease, and so on.^22^ In the UKCTOCS, the 3266 general practices did no cleaning of lists, as we were concerned that this would affect timely mailing of invitations, which was crucial to maintain target recruitment. Thirty two of 1.2 million women invited complained about being contacted. This included four women with ovarian cancer out of an estimated 587 who would have been invited on the basis of national incidences.^23^ Given the small numbers excluded in FLEXISIG, the low rate of complaints in the UKCTOCS, and the substantial effort required, “cleaning up” of local authority lists does not seem to be necessary.

The overall acceptance rate was one in four (23%). However, the rates varied between different parts of the country. The data suggest that in cities such as London, Manchester, and Belfast one should expect lower acceptance rates in the range of one in five. However, in other parts of the country, such as Nottingham, Portsmouth, Bristol, and Derby, almost one in three women in the 50-74 age group might accept an invitation to participate in a screening trial. The varying uptake highlights the importance of running pilots at multiple centres. The UKCTOCS data are being analysed to see whether acceptance rates can be estimated by using age and the postcode derived index for multiple deprivation.

We could not account for undelivered invitations in our trial, as the initial invitations were posted in envelopes with no return address. In the recent FLEXISIG trial, 4% of invites were undelivered,^12^ and this is useful in calculating accurate acceptance rates. The overall acceptance rate in the UKCTOCS was similar to that seen in the colorectal cancer screening trial at Dundee and the WISDOM trial involving estrogen use after the menopause.^24^ ^25^ These rates were substantially higher than the 4.3% acceptance rate reported after a mass mailing of more than 3.4 million in the US systolic hypertension (SHEP) trial.^13^ However, they were much lower than the 55% achieved in the UK FLEXISIG trial. One reason is the way randomisation was approached. In the UKCTOCS, we asked women to help to test a new screening programme for ovarian cancer. We told them that we would need to involve 200 000 women, half of whom would be screened and half would have the usual medical care. In FLEXISIG, participants were asked, “If you were invited to have the bowel cancer screening test, would you take up the offer?” The acceptance rate was based on those who answered “yes.”^22^ This emphasises the importance of wording of the invitations, an aspect sometimes overlooked in clinical trials. Meta-analysis of breast screening trials shows that use of direct contact strategies involving telephone or any type of personal contact can also significantly increase uptake. However, this is only feasible in smaller trials, and concerns about the cost effectiveness have been expressed.^26^ Another way to improve acceptance rates in screening trials is to invite people who regularly attend established screening programmes such as breast and cervical cancer screening, as they are self selected for their belief in screening.^27^ In the Million Women Study, 71% of women having breast screening returned the study questionnaire compared with 53% of all those invited.^28^ This was not possible in the UKCTOCS, as these programmes are limited to women aged below 65 in the UK.

In the UKCTOCS, 202 638 women were randomised in 4.3 years, which translates to a randomisation rate of 47 125 per year. The only trial to report higher rates was the FLEXISIG trial, with a randomisation rate of 68 173 per year.^12^ This was achieved by adopting a two stage recruitment design; 170 483 people who agreed to attend bowel cancer screening if invited were randomised, and only 40 674 allocated to the screen arm were recruited.^29^ This design, however, does mean that only limited data and no biological samples are available in the control group. In addition, recent revisions of laws such as the Data Protection Act and the Mental Capacity Act might lead to difficulties in follow-up through the Office for National Statistics and use of data in secondary studies. None the less, postal recruitment with attendance at the clinic only for people randomised to the study arm is an attractive design with significant savings in terms of time and resources and is definitely worth exploring.

Sophisticated web based trial management systems are becoming the norm for multicentre clinical trials.^29^ ^30^ They enable simultaneous data entry from multiple sites by using standard web browsers with centralised data processing. In large multicentre screening trials such as the UKCTOCS, such technology ensures strict adherence to the protocol over a long period of time. Such solutions can be prohibitively expensive, however, especially for individual research groups. A move to encourage research and funding organisations to combine efforts to produce an open source solution for management of trial data is now occurring. This would empower a wider variety of people to do trials and may encourage more investigators in resource poor settings to take part in high standard research.^31^

A novel feature of the UKCTOCS was the use of an information video and group discussion during recruitment. The video ensured that all participants received high quality standardised information which was sustainable. Research nurses recruiting an average of 100 women a week find it difficult to deliver the same high quality message repeatedly. The video also helped with retention of experienced staff by relieving the monotony of repeating the same message many times a day over four years of recruitment. The group discussions often generated queries that helped volunteers to arrive at a more educated and informed decision than might have been possible if they were only seen individually in a busy recruitment clinic.

One in 6.5 women aged 50-74 in England, Wales, and Northern Ireland (total population 8 031 463) were invited to participate in the UKCTOCS.^32^ This is a major strength, as it makes extrapolation of the findings relevant to people planning randomised controlled trials in the UK. However, the population is not wholly representative of the UK, as the pre-identified Scottish centres were unable to participate.

### Implications of the study

Running large multicentre trials is challenging. Senior investigators need to set aside time to support day to day trial management. Centralisation and automation of trial processes by use of web based trial management systems with high security encryption are essential. Information videos and group discussions allow sustained delivery of high quality standardised information during prolonged recruitment. The over-riding approach needs to be one that incorporates proactive management, flexibility, and individualised solutions. Close cooperation and regular communication between the coordinating and trial centre teams are key to success.

**What is already known on this topic**

In the UK, less than one third of multicentre randomised controlled trials achieve their original recruitment target
Limited attention is paid to the management and conduct of these trials

**What this study adds**

Successful recruitment to trials needs constant monitoring by a committed proactive management team, able to rapidly deliver individual solutions as problems arise
Centralisation and automation of trial processes by use of interactive web based trial management systems are crucial in large multicentre randomised controlled trials
Recruitment can be facilitated by using information videos and group discussions

## Notes

We are particularly grateful to the women throughout the UK who are participating in the trial and to the entire medical, nursing, and administrative staff who work on the UKCTOCS.

Contributors: IJ, UM, MP, SS, SC, LF, and AM were involved in study design and concept. UM, AG-M, AR, SL, TL, KG, DO, JH, KW, MS, IS, TM, RW, JM, SD, NA, SL, and DC were involved in acquisition of data. UM, AG-M, AR, and MB did the statistical analysis. UM, AG-M, AR, and IJ were responsible for interpretation of data. UM, AG-M, AR, and IJ drafted the manuscript. All authors critically revised the manuscript and approved the final version. UM is the guarantor.

Ethical approval: The study was approved by the UK North West Multicentre Research Ethics Committees (North West MREC 00/8/34) with site specific approval from the local regional ethics committees and the Caldicott guardians (data controllers) of the primary care trusts.

Provenance and peer review: Not commissioned; externally peer reviewed.

## Notes

**Cite this as: ***BMJ* 2008;337:a2079
